# In Vivo Comparison of Laser Fluorescence, Near-Infrared Transillumination, and Conventional Methods for Approximal Caries Detection

**DOI:** 10.3390/jcm15187178

**Published:** 2026-09-16

**Authors:** Arda Bingül, Oya Bala, Sinem Akgül, Ceyda Sarı

**Affiliations:** 1 Department of Restorative Dentistry, Faculty of Dentistry, Gazi University, 06510 Ankara, Türkiye; oyabala@gazi.edu.tr (O.B.); sinemakgul@gazi.edu.tr (S.A.); 2 Department of Restorative Dentistry, Faculty of Dentistry, Istanbul Medipol University, 34083 Istanbul, Türkiye; ceyda.gundogdu@medipol.edu.tr

**Keywords:** approximal caries, near-infrared transillumination, laser fluorescence, bitewing radiography, diagnostic accuracy

## Abstract

**Background/Objectives:** The aim of this in vivo study was to compare the diagnostic performance of conventional methods, including visual examination according to the International Caries Detection and Assessment System II (ICDAS II) and digital bite-wing radiography, with laser fluorescence (DIAGNOdent Pen) and near-infrared transillumination (DIAGNOcam and VistaCam iX-Proxi head) for the detection of non-cavitated approximal caries and to evaluate inter-examiner agreement. **Methods:** A total of 76 non-cavitated posterior teeth/surfaces from patients aged 15–60 years were independently assessed by three investigators. Enamel caries validation was based on combined ICDAS II and bite-wing radiographic findings, whereas dentin caries validation was established by cavity opening and direct clinical assessment. Diagnostic performance was evaluated using sensitivity, specificity, predictive values, accuracy, and receiver operating characteristic analysis. Inter-examiner agreement was assessed using the kappa coefficient, and diagnostic performance was compared using the McNemar test (*p* < 0.05). **Results:** Sensitivity ranged from 7.5% for visual examination to 94.3% for DIAGNOdent Pen, whereas specificity ranged from 12.6% to 100.0%. Bite-wing radiography demonstrated the highest diagnostic performance (accuracy: 87.7%; AUC: 0.775), followed by DIAGNOcam (accuracy: 86.0%; AUC: 0.738) and VistaCam (accuracy: 84.2%; AUC: 0.720). Although DIAGNOdent Pen showed high sensitivity, its specificity and overall accuracy were low (31.6%; AUC: 0.535). Inter-examiner agreement was substantial for visual examination, bite-wing radiography, and DIAGNOcam, moderate for VistaCam, and slight for DIAGNOdent Pen. **Conclusions:** Within the limitations of this study, combining visual examination, digital bite-wing radiography, and near-infrared transillumination methods may improve the detection of approximal caries, particularly at an early stage.

## 1. Introduction

Approximal caries, occurring on the contacting surfaces of adjacent teeth, represent one of the most diagnostically challenging conditions in clinical dentistry due to limited accessibility and direct visualization of these regions [1]. The tendency for plaque accumulation in proximal areas, combined with the inherent difficulty of effective oral hygiene, facilitates both the initiation and progression of these lesions [2]. Importantly, approximal caries often remain asymptomatic in the early stages, leading to delayed detection until they extend into dentin, which may require more invasive restorative interventions.

Visual examination is one of the most commonly employed methods for caries detection due to its non-invasive, simple, and cost-effective nature. However, its diagnostic performance for approximal surfaces is limited by restricted visibility, and its accuracy largely depends on the clinician’s experience. Consequently, visual inspection demonstrates relatively low sensitivity for detecting early enamel lesions, and distinguishing early demineralization from sound enamel can be challenging [3,4,5,6]. Therefore, visual examination is commonly complemented by radiographic methods, particularly bite-wing radiography.

Recent consensus recommendations support visual-tactile examination using structured classification systems as the first-choice approach for caries lesion detection and staging on accessible surfaces. Intraoral radiography, preferably bite-wing radiography, is recommended as an adjunctive method when further assessment is indicated. Non-ionizing diagnostic technologies may also provide additional information in selected clinical situations, particularly when early or non-cavitated lesions are suspected [7].

Conventional bite-wing radiography has long been considered the gold standard for detecting approximal caries, as it allows simultaneous visualization of tooth crowns and alveolar bone levels. This facilitates detection of lesions approaching the enamel-dentin junction, with reduced superimposition of proximal surfaces. However, radiographic detection generally requires 30–40% mineral loss, limiting sensitivity for early enamel lesions. Additional drawbacks include two-dimensional imaging, anatomical superimpositions, limited contrast, and exposure to ionizing radiation [1,3,6].

Digital bite-wing radiography has largely replaced conventional film-based systems, providing advantages such as lower radiation dose, immediate image availability, and post-acquisition manipulation (e.g., contrast, brightness, and magnification adjustments). These features may improve the detection of early approximal lesions and provide more objective diagnostic information. Nevertheless, limitations include dependence on sensor resolution, potential artifact formation, reduced patient comfort due to rigid sensors, and higher initial costs. Excessive image manipulation may also increase the risk of false-positive or false-negative interpretations [1,3,6].

The increasing demand for early caries detection and minimally invasive approaches has driven the development of more sensitive, non-invasive diagnostic technologies. Among these, optical and laser-based systems have attracted considerable attention, enabling real-time detection of early demineralization without ionizing radiation [8,9].

Near-infrared (NIR) transillumination systems, such as DIAGNOcam and VistaCam iX-Proxi head, detect carious lesions based on the differing optical properties of sound and demineralized dental tissues. These systems offer radiation-free imaging, non-invasive application, real-time visualization, and the ability to monitor lesion progression [10].

Laser fluorescence devices, such as the DIAGNOdent Pen, detect fluorescence signals emitted by bacterial metabolites in carious tissues. Demineralized areas with bacterial activity produce higher fluorescence signals when exposed to laser light, providing quantitative numerical values. This output enables objective detection and monitoring of carious lesions [11,12,13].

Despite increasing clinical use, the diagnostic performance of optical and laser-based systems for approximal caries remains controversial. Studies comparing these methods with conventional approaches have reported conflicting results [14,15,16]. Furthermore, most current studies were conducted in vitro in a laboratory setting and compared with only a limited number of alternative methods; this may not fully reflect the conditions encountered in clinical use.

In vivo studies directly comparing NIR transillumination and laser fluorescence devices with conventional methods are still limited, especially when all methods are evaluated within the same clinical setting and patient population. Furthermore, the application and interpretation of these systems in the patient’s mouth can be affected by clinician experience and evaluation criteria, and sufficient information on this aspect is lacking.

Therefore, the aim of this in vivo study was to compare the diagnostic performance of conventional methods (visual examination using ICDAS II and digital bite-wing radiography), laser fluorescence (DIAGNOdent Pen), and near-infrared transillumination (NIRT; DIAGNOcam and VistaCam iX-Proxi head) for the detection of non-cavitated approximal caries. The study also evaluated the ability of these methods to identify enamel and dentin lesions, as well as inter-examiner agreement during the diagnostic process.

This study provides a comprehensive clinical comparison of conventional and optical/laser-based diagnostic systems under identical conditions, offering clinically relevant evidence on their reliability and potential for early detection of approximal caries.

Null hypotheses:

1. There is no difference among the tested diagnostic methods in detecting enamel and dentin approximal caries.
2. There is no significant difference between the diagnostic methods evaluated and the reference standard.
3. There is no difference between examiners in detecting enamel and dentin caries for each diagnostic method.

## 2. Materials and Methods

### 2.1. Study Design and Ethical Approval

This prospective diagnostic accuracy study was conducted after obtaining ethical approval from the Medipol University Ethics Committee (Approval No: 2023/536). The study was designed to evaluate and compare the diagnostic accuracy of different caries detection methods against predefined reference standards. As participants were not prospectively assigned to a health-related intervention for the purpose of evaluating its effect on a health outcome, the study did not meet the ICMJE definition of a clinical trial and was therefore not prospectively registered in a clinical trial registry. Sample size was determined using G*Power (Version 3.1.9.6) with a 95% confidence level, 80% statistical power, and an effect size of 0.50. The analysis indicated that at least 63 teeth/surfaces were required. To minimize potential errors, 76 teeth/surfaces with suspected approximal caries were included in the study.

All patient evaluations and treatment procedures were performed at the Department of Restorative Dentistry, Gazi University Faculty of Dentistry.

### 2.2. Inclusion Criteria

Participants were included in the study according to the following criteria:

1. Systemically healthy individuals classified as ASA I according to the American Society of Anesthesiologists;
2. Age between 15 and 60 years;
3. Absence of fixed prosthetic restorations on the evaluated tooth or adjacent teeth;
4. Not undergoing fixed orthodontic treatment;
5. Absence of crowding that could cause anatomical variation;
6. No systemic disease that could affect oral flora;
7. No missing teeth or restorative treatment requirement in the mesial or distal approximal contact areas;
8. Periodontally healthy condition;
9. Absence of cavitation detected during visual examination;
10. The evaluated approximal surfaces were restricted to posterior-to-posterior contacts and included the distal surfaces of permanent first premolars (14, 24, 34, and 44), the mesial and distal surfaces of permanent second premolars (15, 25, 35, and 45), the mesial and distal surfaces of permanent first molars (16, 26, 36, and 46), and the mesial surfaces of permanent second molars (17, 27, 37, and 47). Tooth numbers are reported according to the FDI tooth numbering system. This selection ensured that all evaluated approximal surfaces represented contacts between posterior teeth and reduced anatomical variability associated with anterior-to-posterior proximal contacts.

All participants were informed about the study procedures and diagnostic methods, and written informed consent was obtained.

Prior to examination, plaque and surface discoloration were removed using a fluoride-free prophylaxis paste (Klint, Voco, Düsseldorf, Germany) with a prophylactic rubber cup. Subsequently, surfaces with suspected approximal caries were evaluated using five diagnostic methods: visual examination, digital bite-wing radiography, near-infrared light transillumination devices (DIAGNOcam (KaVo Dental GmbH, Biberach, Germany) and VistaCam iX-Proxi head (Dürr Dental SE, Bietigheim-Bissingen, Germany)), and a laser fluorescence device (DIAGNOdent Pen (KaVo Dental GmbH, Biberach, Germany)).

All assessments were performed independently and in random order by three investigators from the Department of Restorative Dentistry with clinical experience of 5, 16, and 39 years, respectively. Prior to the study, the investigators received theoretical information and practical training on the diagnostic methods using patients who were not included in the study. All evaluations were conducted under standardized lighting conditions and using the same computer monitor. The evaluation criteria of the diagnostic methods tested in the study are presented in Table 1.

### 2.3. Evaluation by Visual Examination

For visual examination, tooth surfaces were air-dried and evaluated under dental unit light using a mirror and probe according to the ICDAS II criteria [17] (Table 1). Subsequently, the obtained scores were classified as 0 for sound surfaces, 1–3 for enamel lesions, and 4–6 for dentin lesions.

### 2.4. Evaluation by Digital Bite-Wing Radiography

Digital bite-wing radiographs were obtained using a radiographic device (CCX, Tropy, UK) operating at 70 kVp and 1.12 mAs. A film holder supporting the paralleling technique was used to standardize image acquisition. A size-2 phosphor plate (VistaScan phosphor plate No. 2, Dürr Dental SE, Bietigheim-Bissingen, Germany) was positioned appropriately for the relevant tooth/teeth. The images were evaluated according to the criteria presented in Table 1 [19,20]. For diagnostic accuracy analyses, radiographic score 0 was classified as sound, scores 1–2 as enamel lesions, and scores 3–4 as dentin lesions.

### 2.5. Evaluation by DIAGNOcam

The teeth were air-dried, and the oral environment was isolated using cotton rolls and a saliva ejector. The intraoral camera probe was positioned in contact with the vestibular and lingual/palatal surfaces of the tooth in accordance with the manufacturer’s instructions, and images were obtained in a fixed position. The captured images were evaluated according to the criteria presented in Table 1 [21]. During the evaluation, brightness and contrast were adjusted using the device software. Subsequently, for DIAGNOcam, a score of 0 was classified as sound, scores of 1–3 as enamel lesions, and scores of 4–5 as dentin lesions.

### 2.6. Evaluation by VistaCam iX-Proxi Head

The surfaces of the teeth were air-dried and isolated from the oral environment using cotton rolls and a saliva ejector. The probe was positioned close to the approximal surface according to the manufacturer’s instructions, and images were obtained with the aid of a spacer. The images were evaluated according to the criteria presented in Table 1 [22]. Brightness and contrast adjustments were performed using the software when necessary. Following this, Score 1 was classified as an enamel lesion, whereas Score 2 was classified as a dentin lesion.

### 2.7. Evaluation by DIAGNOdent Pen

Before clinical use, the device was calibrated using the calibration applicator according to the manufacturer’s instructions and recalibrated for each new tooth surface. The relevant surface was air-dried and isolated using cotton rolls or a saliva ejector. The wedge-shaped tip was positioned in the gingival embrasure, and the approximal surface was scanned using circular movements to obtain numerical values displayed on the digital screen. Each surface was measured three times, and the arithmetic mean was calculated; this value was recorded as the DIAGNOdent Pen score for the respective tooth. The scores were interpreted according to the criteria presented in Table 1 [18]. DIAGNOdent Pen values were classified based on the manufacturer’s recommended threshold values. Digital readings between 0 and 6 were considered sound, values between 6.1 and 15 were classified as enamel lesions, and values above 15 were classified as dentin lesions.

### 2.8. Clinical Evaluation

For clinical evaluation, before determining the final diagnostic status of the teeth/surfaces included in the study, all cases were re-examined in a separate session by clinical visual inspection, and bite-wing radiographs were reassessed. Three examiners independently evaluated the teeth/surfaces, and the final diagnostic assessment was established by consensus [23]. The reference standard was defined separately according to lesion depth.

For enamel caries, the reference standard was established based on a combination of visual examination according to the ICDAS II criteria and digital bite-wing radiographic findings [24,25]. These assessments were used specifically to establish the reference standard rather than as an additional diagnostic modality to be compared with the evaluated methods. The combination of clinical and radiographic findings was used because approximal enamel caries may not always be reliably identified by visual examination alone. In cases where consensus regarding enamel caries was reached, cavity opening was not performed for ethical reasons; instead, appropriate preventive measures and follow-up were recommended.

For dentin caries, the reference standard was based on direct clinical assessment following cavity opening performed by a single examiner [16,23,24,25]. Cavity preparation was initially performed using rotary instruments and diamond burs, during which the presence and localization of the carious lesion (enamel or dentin) were recorded. For dentin lesions, the depth and hardness of the carious tissue were assessed using a periodontal probe, and infected dentin was removed using diamond burs and hand excavators until only hard dentin remained [16]. The subsequent restoration of cavities opened for clinical assessment was performed for ethical and clinical management purposes and was not considered a diagnostic criterion or component of the reference standard.

The final outcome was determined by scoring each tooth/surface according to lesion severity. After cavity preparation, the presence and extent of caries were classified as follows: Score 0, sound; Score 1, enamel caries; Score 2, dentin caries; and Score 3, deep dentin caries extending to the pulp [26]. For the diagnostic analyses, Scores 2 and 3 were classified as dentin caries (cut-off ≥ 2).

Following caries removal, cavities were isolated with a rubber dam. Enamel surfaces were selectively etched with 37% orthophosphoric acid (ProEtch, Promida, Izmir, Turkey) for 30 s, rinsed, and gently air-dried. A universal adhesive (Scotchbond Universal, 3M, Eagan, MN, USA) was applied according to the manufacturer’s instructions. Cavities were restored using a universal resin composite (Filtek Universal Restorative, 3M, St. Paul, MN, USA) placed in 2 mm incremental layers. Finishing and polishing procedures were performed using aluminum oxide–coated discs (Sof-Lex, 3M ESPE, St. Paul, MN, USA).

To verify lesion depth after restoration, digital bite-wing radiographs were obtained using the paralleling technique. Pre- and post-restorative radiographs were digitally superimposed to assess lesion extent and depth, thereby confirming the clinical scoring.

### 2.9. Statistical Analysis

Statistical analyses were performed using SPSS software (Version 26.0, IBM Corp., Armonk, NY, USA), with a significance level set at 0.05 and a 95% confidence interval. For each diagnostic method, sensitivity, specificity, positive predictive value (PPV), negative predictive value (NPV), and accuracy were calculated and reported with their corresponding 95% confidence intervals.

The diagnostic performance of the methods was assessed using receiver operating characteristic (ROC) analysis. ROC curves were generated using the original ordinal or continuous scores obtained before the data were dichotomized into caries present/absent.

Agreement between the diagnostic methods and the reference-standard reference was evaluated using the McNemar test, and inter-examiner agreement was assessed using the Fleiss Kappa coefficient.

## 3. Results

**Figure 1:**
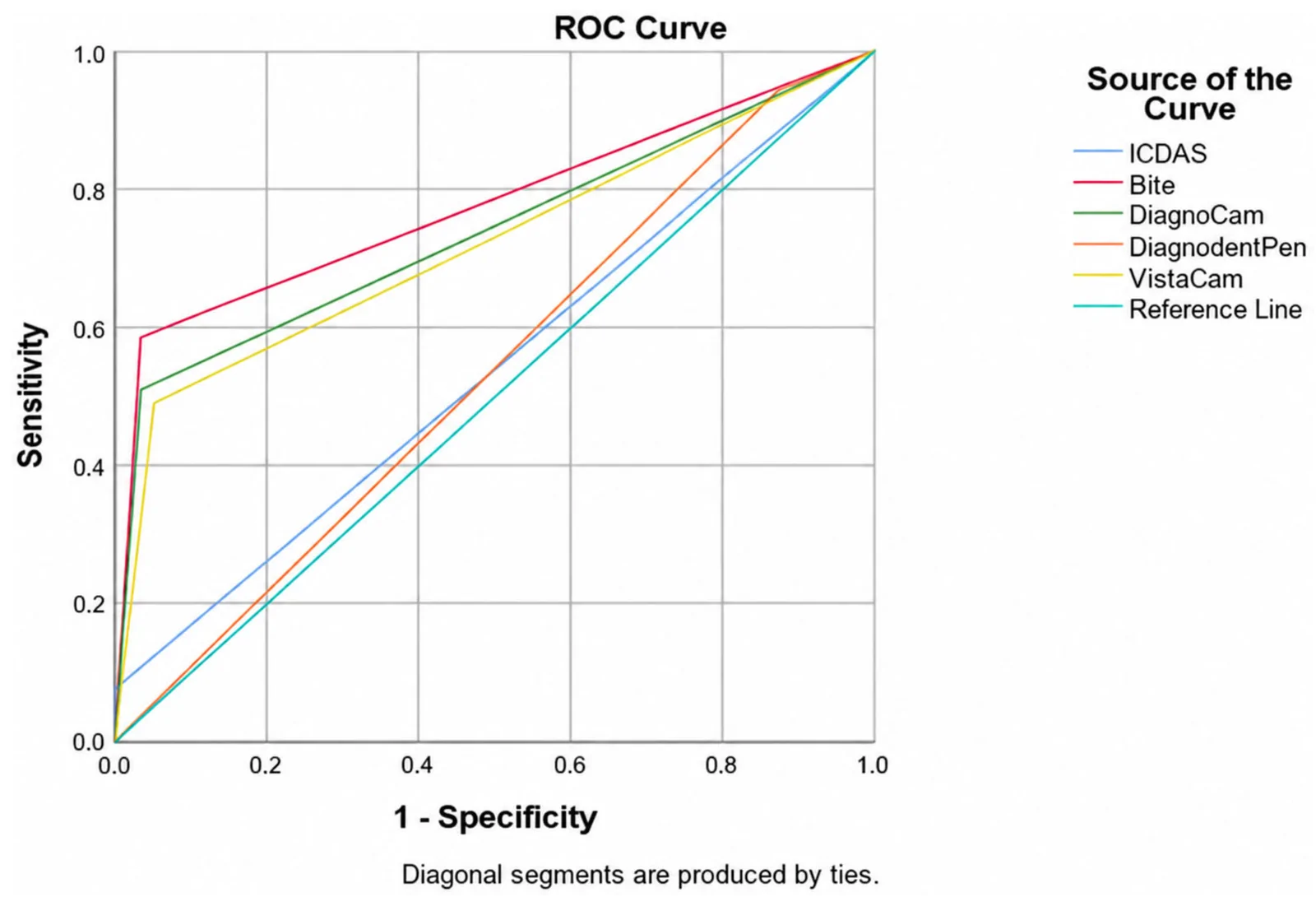
Receiver Operating Characteristic (ROC) analysis comparing the diagnostic performance of different caries detection methods.

The Receiver Operating Characteristic (ROC) analysis graph of the caries detection methods evaluated in this study is presented in Figure 1. The sensitivity, specificity, positive predictive value, negative predictive value, accuracy, and area under the ROC curve (AUC) values obtained for each tested caries detection method are shown in Table 2.

Different sensitivities, specificities, positive and negative predictive values, accuracy rates, and AUC values were obtained for each of the tested caries-detection methods. The lowest sensitivity was observed with visual examination, whereas the highest sensitivity was obtained with the DIAGNOdent Pen. Except for the DIAGNOdent Pen, the specificity values of the caries-detection methods were similar. The lowest positive predictive values were obtained with the DIAGNOdent Pen. Negative predictive values were similar across all test methods. The accuracy rates of the caries detection methods were also similar, except for the DIAGNOdent Pen. When the AUC values were examined, those for visual examination and DIAGNOdent Pen were lower than those for the other caries detection methods (Table 2).

### 3.1. Comparison of the Effectiveness of the Caries Detection Methods in Identifying Enamel and Dentin Carious Lesions

The findings on the effectiveness of the caries-detection methods in identifying enamel and dentin caries are presented in Table 3.

A total of 76 teeth/surfaces were evaluated in the study. Visual examination identified 43 enamel and 6 dentin caries; digital bite-wing radiography identified 63 enamel and 13 dentin caries; DIAGNOcam identified 21 enamel and 26 dentin caries; VistaCam iX-Proxi head identified 29 enamel and 13 dentin caries; and DIAGNOdent Pen identified 4 enamel and 72 dentin caries.

In detecting enamel lesions, the highest sensitivity (100%) and accuracy (98.9%) were obtained with visual examination. This was followed by DIAGNOcam (98.3% sensitivity and 97.1% accuracy), digital bite-wing radiography (97.7% sensitivity and 96.6% accuracy), and VistaCam iX-Proxi head (94.8% sensitivity and 93.7% accuracy). The lowest sensitivity (12.8%) and accuracy (13.8%) were obtained with the DIAGNOdent Pen.

In detecting dentin lesions, the highest diagnostic performance was achieved with the DIAGNOdent Pen, with 94.1% sensitivity, 94.1% positive predictive value, and 88.9% accuracy. Digital bite-wing radiography demonstrated 60.8% sensitivity and 57.4% accuracy; DIAGNOcam demonstrated 52.9% sensitivity and 50.0% accuracy; VistaCam iX-Proxi head demonstrated 51.0% sensitivity and 48.1% accuracy; and visual examination demonstrated 7.8% sensitivity and 7.4% accuracy (Table 3).

### 3.2. Evaluation of the Agreement Between Caries Detection Methods and the Reference Standard

**Figure 2:**
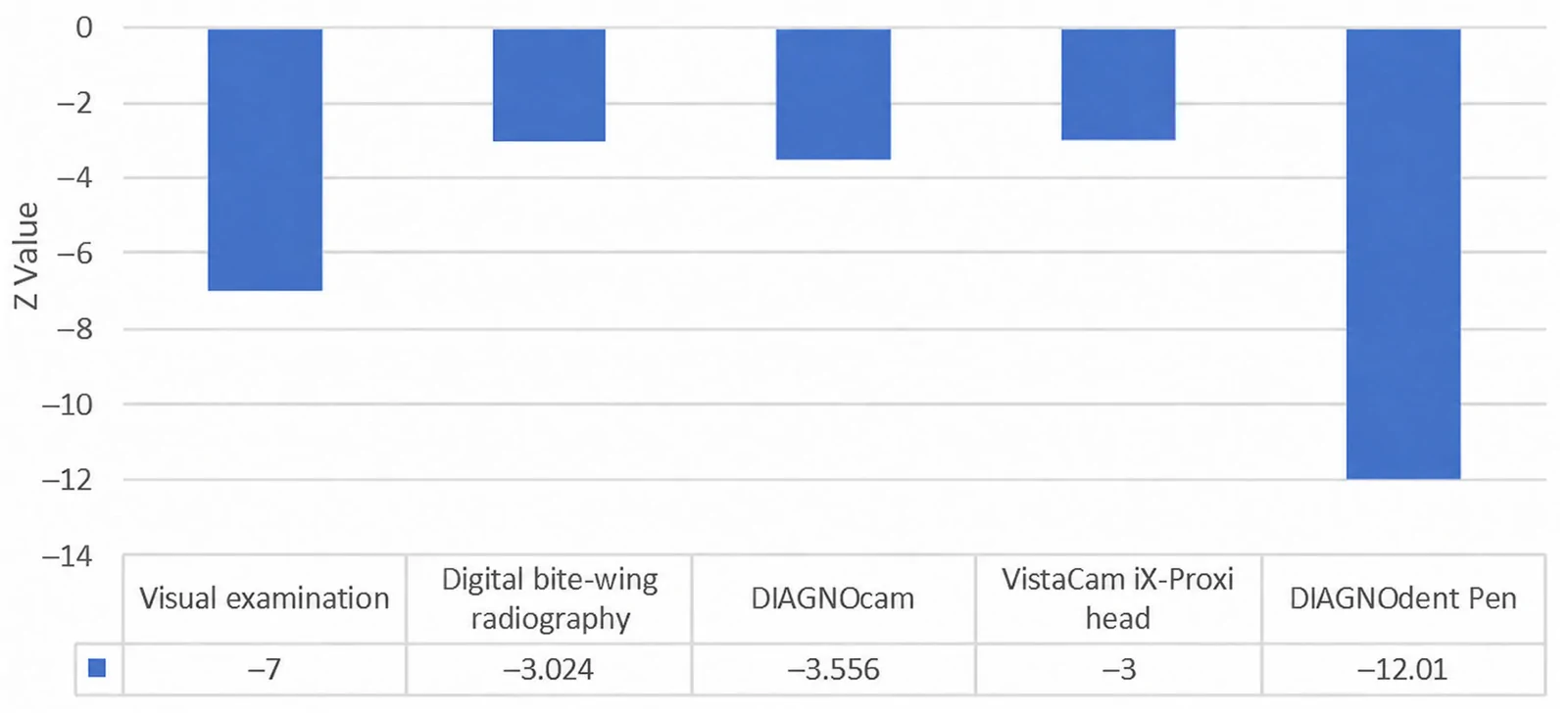
Comparison of caries detection methods with the reference standard.

Statistically significant differences were found between the caries detection methods and the reference standard (*p* < 0.05) (Figure 2). A highly significant disagreement was observed between visual examination and the reference standard (Z = −7.000, *p* < 0.001). Significant disagreement with the reference standard was also detected for digital bite-wing radiography (Z = −3.024, *p* = 0.002), DIAGNOcam (Z = −3.536, *p* < 0.001), and VistaCam iX-Proxi head (Z = −3.000, *p* = 0.003). A very strong level of disagreement was observed between the DIAGNOdent Pen and the reference standard (Z = −12.010, *p* < 0.001).

### 3.3. Evaluation of Inter-Examiner Agreement

**Figure 3:**
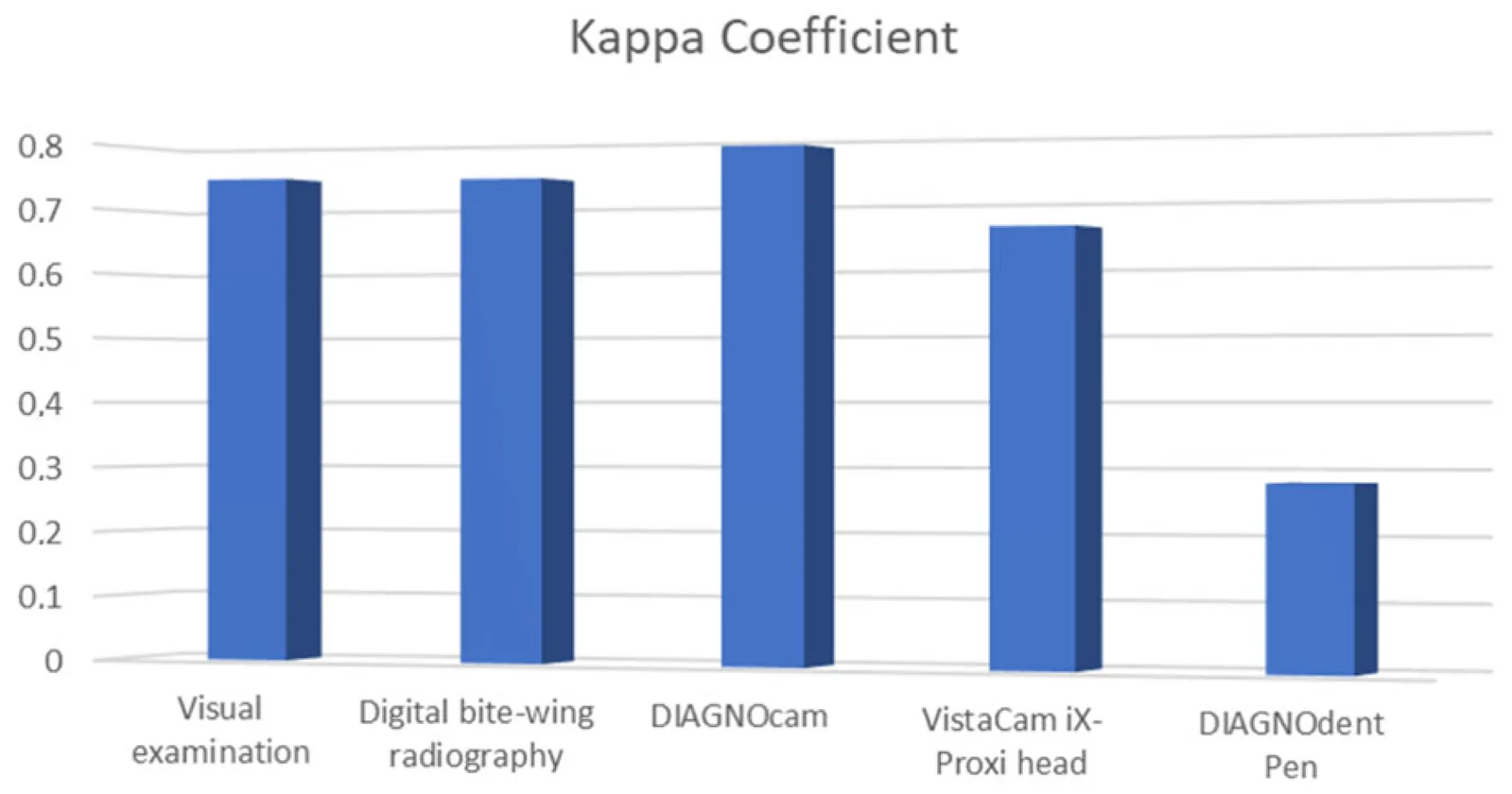
Graphical representation of inter-examiner agreement for each caries detection method.

The findings regarding inter-examiner agreement for the caries detection methods are presented in Table 4 and Figure 3.

Inter-examiner agreement was assessed using Fleiss’ kappa coefficient. The calculated kappa values indicated good agreement for visual examination, bite-wing radiography, and DIAGNOcam, moderate agreement for VistaCam iX-Proxi head, and fair agreement for DIAGNOdent Pen.

For visual examination, the rate of diagnosing carious lesions in enamel was very high among the investigators (98.7% for the 1st and 3rd investigators, 97.4% for the 2nd investigator). However, the rate of detecting dentin carious lesions was very low (below 2.6%). The kappa coefficient calculated for this method was 0.746.

With digital bite-wing radiography, investigators detected enamel carious lesions at rates ranging from 81.6% to 85.5%, and dentin lesions at rates ranging from 14.5% to 18.4%. The kappa coefficient for this method was calculated as 0.742.

Using DIAGNOcam, enamel carious lesions were detected at rates of 84.2–86.8%, and dentin lesions at 13.2–15.8%. The kappa coefficient for this method was 0.787.

With the VistaCam iX-Proxi head, the detection rates for enamel carious lesions ranged from 82.9% to 88.2%, and for dentin lesions from 11.8% to 17.1%. The kappa coefficient for this method was 0.662.

With the DIAGNOdent Pen, the detection rate of enamel carious lesions ranged from 63.9% to 19.7%. For dentin lesions, the first investigator detected caries at a rate of 90.8%, the second at 96.1%, and the third at 80.3%. The kappa coefficient for this method was calculated as 0.281.

## 4. Discussion

In the present study, the diagnostic performance of various caries detection methods was evaluated using sensitivity, specificity, positive predictive value (PPV), negative predictive value (NPV), accuracy, and area under the receiver operating characteristic (ROC) curve (AUC).

Visual examination demonstrated very low sensitivity (7.5%) but perfect specificity (100%) and PPV (100%), with acceptable NPV (78.1%) and accuracy (78.5%). The low AUC value (0.538) further indicates its limited discriminative ability. These findings clearly demonstrate that visual examination alone is insufficient to detect non-cavitated approximal lesions, which often progress beneath an intact enamel surface without visible clinical changes. Conversely, its high specificity reflects a tendency to classify only clearly visible lesions as carious, thereby minimizing false-positive results.

Although the use of ICDAS II improves the standardization of visual assessment and supports minimally invasive treatment planning [27], its effectiveness remains limited on approximal surfaces due to restricted visibility and variability in clinical conditions such as lighting, moisture control, and surface dryness [28,29,30].

Digital bite-wing radiography demonstrated moderate sensitivity, high specificity, PPV, and NPV, along with higher accuracy and AUC than visual examination. These findings indicate that bite-wing radiography provides more reliable discrimination between sound and carious approximal surfaces. Recent systematic evidence similarly indicates that bite-wing radiography generally demonstrates good diagnostic performance for proximal caries, although diagnostic estimates vary across studies and clinical evidence remains limited. Differences in study design, diagnostic thresholds, and reference standards have been identified as important sources of heterogeneity and potential bias in proximal caries diagnostic studies.

However, the diagnostic performance of bite-wing radiography is also influenced by exposure parameters and imaging conditions. In accordance with the ALARA principle, standardized acquisition settings are essential to ensure optimal image quality while minimizing radiation exposure [31,32,33]. In the present study, this was achieved by using fixed exposure parameters and a paralleling technique.

Near-infrared light transillumination systems (DIAGNOcam and VistaCam iX-Proxi head) demonstrated moderate sensitivity and good specificity, along with satisfactory accuracy and AUC values. The moderate sensitivity may be attributed to increased light scattering and absorption in deeper lesions, particularly those involving dentin, whereas the high specificity indicates a low probability of false-positive findings. Recent systematic evidence has similarly shown considerable variability in the diagnostic performance of near-infrared technologies under clinical conditions. Elsawaf et al. reported wide ranges of sensitivity and specificity across studies and indicated that lesion location and extent, differences in scoring approaches, reference standards, study design, and the devices used may influence the reported diagnostic performance of these systems [34]. DIAGNOcam enables visualization of the enamel–dentin junction, facilitating the differentiation between enamel and dentin level lesions. Previous studies have reported that although its sensitivity may be lower than that of bite-wing radiography, its diagnostic performance for dentin caries is comparable [8,23,35,36], findings consistent with the present study. More recently, Wang et al. compared DIAGNOcam with ICDAS and bite-wing radiography for proximal caries detection in permanent teeth. DIAGNOcam showed significantly higher sensitivity than both visual examination and bite-wing radiography at the early enamel threshold, whereas bite-wing radiography showed the highest AUC at the dentin threshold, although the differences among the methods were not statistically significant. These findings suggest that the relative diagnostic performance of DIAGNOcam may vary according to lesion depth [37]. Previous studies have also reported that although the sensitivity of DIAGNOcam may be lower than that of bite-wing radiography, its diagnostic performance for dentin caries is comparable [8,23,35,36], which is consistent with the findings of the present study.

The performance of the VistaCam iX-Proxi head may be particularly influenced by clinical variables. Factors such as surface moisture, probe angulation, and lesion location may affect image quality and interpretation [18,38]. In addition, artifacts and limited visualization of certain approximal regions may reduce diagnostic reliability [39,40]. More recently, Edrees et al. clinically evaluated the VistaCam iX-Proxi head for proximal caries detection and reported high sensitivity but comparatively lower specificity when its findings were compared with ICDAS II and bite-wing radiography [41]. The variability between these findings and those reported in other studies may be related to differences in lesion position and extent, as well as methodological differences and the reference standards used for comparison. These factors may help explain the relatively lower diagnostic performance of the VistaCam iX-Proxi head observed in the present study compared with DIAGNOcam and bite-wing radiography.

The laser fluorescence device DIAGNOdent Pen showed high sensitivity and NPV but low specificity, PPV, accuracy, and AUC. While high sensitivity suggests the device is effective at ruling out sound surfaces, its low specificity indicates a high rate of false-positive findings. This is likely due to fluorescence signals generated by non-carious factors such as plaque, staining, calculus, and hypomineralization [42], leading to potential overestimation of caries presence.

The performance of the DIAGNOdent Pen also varies depending on lesion depth and bacterial activity. While early enamel lesions produce weak fluorescence signals, dentin lesions, which exhibit higher bacterial activity and porphyrin accumulation, generate stronger signals [43,44]. This may explain the higher sensitivity observed for dentin caries in the present study. However, limitations related to probe positioning and restricted access to approximal surfaces may adversely affect measurement accuracy. Recent systematic evidence further suggests that the diagnostic performance of laser fluorescence for proximal caries detection may vary according to study conditions and diagnostic thresholds. Janjic Rankovic et al. reported favorable diagnostic performance for laser fluorescence, particularly in laboratory studies, while emphasizing that the available clinical evidence remains limited [45]. Similarly, a systematic review and meta-analysis by Serban et al. identified considerable heterogeneity among studies evaluating non-ionizing diagnostic technologies. For laser fluorescence, heterogeneity was particularly high in proximal-surface subgroups, for which the available evidence remained inconclusive [46]. These findings highlight the influence of study conditions and diagnostic thresholds on the reported performance of laser fluorescence and may partly explain the variability between the diagnostic performance observed in the present study and that reported in previous investigations.

The ability of the tested methods to differentiate between enamel and dentin lesions varied considerably. Visual examination showed better performance for enamel lesions, likely because superficial changes such as opacity and discoloration are detectable under optimal conditions, whereas the DIAGNOdent Pen demonstrated superior performance in detecting dentin lesions due to its sensitivity to bacterial by-products. Accordingly, the first null hypothesis, that there is no difference among the tested diagnostic methods in detecting approximal caries in enamel and dentin, was clearly rejected based on the observed differences in diagnostic performance.

Radiographic detection of enamel lesions is known to require a certain degree of mineral loss, which may explain why early non-cavitated lesions are often underestimated [47,48]. Similarly, near-infrared transillumination techniques may have limited contrast in superficial demineralization, thereby reducing their sensitivity for early enamel lesions.

The comparison with the reference standard revealed statistically significant differences for all tested methods, indicating that none of the individual techniques fully matched the diagnostic performance of the combined approach. This finding highlights the limitations of relying on a single diagnostic method in clinical practice. In particular, the discrepancy between visual examination and the reference standard underscores its limited sensitivity, whereas the disagreement observed for other methods reflects their inherent technical and interpretative limitations. Therefore, the second null hypothesis, which assumed no significant difference between the evaluated diagnostic methods and the reference standard, was clearly rejected, as statistically significant discrepancies were identified for all tested methods.

Inter-examiner reliability analysis demonstrated that diagnostic outcomes were influenced not only by the method used but also by clinician-related factors. Visual examination, digital radiography, and DIAGNOcam showed good agreement, whereas DIAGNOdent Pen exhibited poor agreement, suggesting higher operator dependency. These findings emphasize the importance of clinician training, calibration, and standardized protocols in improving diagnostic consistency. In line with these findings, the third null hypothesis, stating that there is no difference between examiners in detecting enamel and dentin caries for each diagnostic method, was also clearly rejected due to the significant inter-examiner variability observed.

Overall, the results of this study indicate that no single method is sufficient for the accurate diagnosis of approximal caries. Visual examination and radiographic methods provided more consistent diagnostic performance, whereas advanced technologies may offer additional diagnostic information but appear to be more susceptible to variability. Therefore, adjunctive diagnostic technologies may be most appropriately interpreted as complementary to conventional clinical and radiographic assessment rather than as standalone replacements. This interpretation is consistent with recent ORCA–EFCD consensus recommendations, which identify visual examination as the first-choice method for caries lesion detection on accessible surfaces, recommend intraoral radiography—preferably bite-wing radiography—as an additional method, and recognize that adjunct non-ionizing technologies may be useful in selected clinical situations [7].

Further studies are needed to evaluate the influence of clinical variables, device settings, and operator experience on the diagnostic performance of caries detection methods.

An important limitation of this study is the reference standard used for enamel lesions. Because operative verification of non-cavitated enamel lesions is not ethically feasible, a combined reference standard based on ICDAS II visual examination and digital bite-wing radiography was applied. However, this approach may introduce partial overlap between the reference standard and some of the evaluated diagnostic methods, potentially limiting full methodological independence. Therefore, the reported diagnostic accuracy for early enamel lesions should be interpreted with caution. Future studies should consider more independent reference standards, such as longitudinal follow-up and/or alternative validation strategies.

## 5. Conclusions

Within the limitations of this in vivo study, the diagnostic performance of caries-detection methods varied by lesion depth and detection principle. Visual examination showed high specificity but low sensitivity for non-cavitated approximal lesions, while digital bite-wing radiography and DIAGNOcam provided more balanced and reliable outcomes. DIAGNOdent Pen demonstrated high sensitivity for dentin lesions but low specificity, limiting its standalone use.

These findings indicate that no single method is sufficient for accurate detection and staging of approximal caries. A combined diagnostic approach, integrating visual, radiographic, and adjunctive optical methods, is recommended to improve diagnostic accuracy and inform minimally invasive treatment decisions.

The observed inter-examiner variability emphasizes the importance of clinician training, calibration, and careful selection of diagnostic devices to ensure consistent, reliable outcomes in clinical practice.

## Figures and Tables

**Table 1:** Evaluation criteria of the diagnostic methods tested in the study [17,18,19,20,21,22].

**ICDAS II Criteria**			
Threshold values	Scores	Diagnostic Criteria	
D0	0	Sound	
D1 (Enamel lesion)	1	First visual change in enamel	
	2	Distinct visual change in enamel	
	3	Localized enamel breakdown (without clinically visible signs of dentin involvement)	
D2 (Dentin lesion)	4	Underlying dark shadow from dentin	
	5	Distinct cavity with visible dentin involvement	
	6	Extensive distinct cavity with visible dentin involvement	
**Evaluation Criteria for Digital Bite-Wing Radiography**			
Threshold values	Scores	Category	Diagnostic Criteria
D0	0	Sound	No radiolucency or restoration
D1 (Enamel lesion)	1	Enamel lesion located in the outer 1/2 of the enamel	Increased radiolucency limited to the outer half of the enamel
	2	Enamel lesion located in the inner 1/2 of the enamel	Increased radiolucency involving both inner and outer halves of enamel without crossing the enamel-dentin junction
D2 (Dentin lesion)	3	Dentin lesion located in the outer 1/2 of the dentin	Radiolucency crossing the enamel-dentin junction but limited to the outer half of dentin
	4	Dentin lesion located in the inner 1/2 of the dentin	Radiolucency extending into the inner half of dentin; the pulp may or may not be involved
**Evaluation Criteria for DIAGNOcam**			
Threshold values	Scores	Definition	Diagnostic and Therapeutic Recommendations
D0	0	Sound surface	Caries monitoring; no active treatment recommended
D1 (Enamel lesion)	1	First visible signs of enamel caries	Caries monitoring; preventive care recommended
	2	Established enamel caries lesion	Caries monitoring; preventive care recommended
	3	Enamel caries reaching the DEJ	Caries monitoring; preventive care recommended
D2 (Dentin lesion)	4	Dentin caries; lesion linearly crossing the DEJ	Bite-wing radiography; minimally invasive operative treatment recommended
	5	Deep dentin caries	Bite-wing radiography; operative treatment recommended
**Evaluation Criteria for Images Obtained with VistaCam iX-Proxi Head**			
	Scores	Diagnostic criteria	
D0	0	No change in enamel	
D1 (Enamel lesion)	1	Wide, bright band with wedge-shaped structures in dark translucent enamel. The lesion may extend to the enamel-dentin junction	
D2 (Dentin lesion)	2	Wide, bright band with wedge-shaped structures crossing the enamel-dentin junction	
**Evaluation Criteria for Scores Obtained with DIAGNOdent Pen**			
Threshold values	Scores	Diagnostic Criteria	
D0	0–6	Sound	
D1 (Enamel lesion)	6.1–9	Caries lesion in the outer half of the enamel	
	9.1–15	Caries lesion in the inner half of the enamel	
D1 (Dentin lesion)	>15	Caries lesion in dentin	

**Table 2:** Sensitivity, specificity, positive and negative predictive values, accuracy rates, and AUC values obtained from caries diagnostic methods.

Caries Diagnostic Methods	Sensitivity(%)	Specificity (%)	PPV (%)	NPV (%)	Accuracy (%)	AUC
Visual examination	7.5	100.0	100.0	78.1	78.5	0.538
Bite-wing radiography	58.5	96.6	83.8	88.5	87.7	0.775
DIAGNOcam	50.9	96.6	81.8	86.7	86.0	0.738
VistaCam iX-Proxi head	49.1	94.9	74.3	86.0	84.2	0.720
DIAGNOdent Pen	94.3	12.6	24.6	88.0	31.6	0.535

**Table 3:** Performance findings of the caries diagnostic methods in detecting enamel and dentin caries.

	Caries Diagnostic Methods	Sensitivity (%)	Specificity (%)	PPV (%)	NPV (%)	Accuracy (%)
**Enamel**	Visual examination	100.0	0.0	98.9	0.0	98.9
	Bite-wing radiography	97.7	0.0	98.8	0.0	96.6
	DIAGNOcam	98.3	0.0	98.8	0.0	97.1
	VistaCam iX-Proxi head	94.8	0.0	98.8	0.0	93.7
	DIAGNOdent Pen	12.8	100.0	100.0	1.3	13.8
**Dentin**	Visual examination	7.8	100.0	100.0	6.0	7.4
	Bite-wing radiography	60.8	33.3	93.9	4.8	57.4
	DIAGNOcam	52.9	0.0	90.0	0.0	50.0
	VistaCam iX-Proxi head	51.0	100.0	100.0	10.7	48.1
	DIAGNOdent Pen	94.1	0.0	94.1	0.0	88.9

**Table 4:** Kappa coefficient values for inter-examiner agreement in each tested caries diagnostic method.

Caries Diagnostic Methods	Researchers	Enamel		Dentin	
		*n*	%	*n*	%
Visual examination	1st Researcher	75	98.7	1	1.3
	2nd Researcher	74	97.4	2	2.6
	3rd Researcher	75	98.7	1	1.3
	Kappa coefficient	0.746			
	*p*	0.000			
Digital bite-wing radiography	1st Researcher	62	81.6	14	18.4
	2nd Researcher	64	84.2	12	15.8
	3rd Researcher	65	85.5	11	14.5
	Kappa coefficient	0.742			
	*p*	0.000			
DIAGNOcam	1st Researcher	66	86.8	10	13.2
	2nd Researcher	65	85.5	11	14.5
	3rd Researcher	64	84.2	12	15.8
	Kappa coefficient	0.787			
	*p*	0.000			
VistaCam iX-Proxi head	1st researcher	63	82.9	13	17.1
	2nd researcher	67	88.2	9	11.8
	3rd researcher	63	82.9	13	17.1
	Kappa coefficient	0.662			
	*p*	0.000			
DIAGNOdent Pen	1st researcher	7	9.2	69	90.8
	2nd researcher	3	3.9	73	96.1
	3rd researcher	15	19.7	61	80.3
	Kappa coefficient	0.281			
	*p*	0.000			

## Data Availability

The data presented in this study are available from the corresponding author upon reasonable request. The data are not publicly available due to privacy and ethical restrictions.

## Abbreviations

The following abbreviations are used in this manuscript:

ALARA	As Low As Reasonably Achievable
ASA	American Society of Anesthesiologists
AUC	Area Under the Receiver Operating Characteristic Curve
DEJ	Dentinoenamel Junction
ICDAS II	International Caries Detection and Assessment System II
NIR	Near-Infrared
NIRT	Near-Infrared Transillumination
NPV	Negative Predictive Value
PPV	Positive Predictive Value
ROC	Receiver Operating Characteristic
SPSS	Statistical Package for the Social Sciences
